# Supplementary material for: “I got courage from knowing that even a daughter-in-law can earn her living”: Mixed methods evaluation of a family-centred intervention to prevent violence against women and girls in Nepal
Source: PLoS One. 2020 May 19;15(5):e0232256. doi: 10.1371/journal.pone.0232256 (PMC7237029; doi:10.1371/journal.pone.0232256)
Supplement: Attachment — Submitted filename: PONE-D-19-25458 Referee comments- Seff.docx [file pone.0232256.s001.docx]

Thank you for providing me with the opportunity to review this article. The authors discuss the evaluation of three-pronged intervention to reduce violence against women and improve their economic well-being. The article is well-written and easy to follow, and the findings are important to share with the development community. However, in addition to the areas for improvement outlined below, I am concerned with the lack of attention paid to the negative outcomes observed in the findings. The authors conclude, “*Our findings provide considerable support for the Sammanit Jeevan whole family approach to reducing intimate partner violence, changing inequitable gender norms and economically empowering young women”,* yet *several* adverse effects were also observed, such as a decline in self-reported health, increase in suicide ideation for older women, increase in frequency of quarreling (as reported by women), increase in IPV for older women, increase in sexual violence perpetration by men, among several others. In addition to addressing the comments below, I would highly recommend that the authors devote considerable attention to these negative findings in the discussion section (in addition to amending the conclusion given above).

**Background**

1. The third paragraph does an excellent job of explicating the factors contributing to young married women’s experiences of violence as perpetrated by their mothers-in-law and how these factors are linked to gender inequality and patriarchal family structures. However, the majority of the Background is focused on this topic, which is not the primary outcome of the intervention as stated in Table 1. I believe the second paragraph could be strengthened by expanding on the mechanisms that foster IPV in this setting, rather than simply stating various characteristics found to be associated with IPV in Nepal.
2. Given that primary outcomes also include economic- and income-based indicators, the Background section should provide background on women’s labor force participation and income decision-making in this context, including barriers to labor participation and the underlying gendered power dynamics that inherently marginalize women in this area. Such discussion of this literature will make clear for the reader why the intervention includes the three primary components of gender transformative norms, economic empowerment, and IGAs support.
3. What evidence currently exists around the effectiveness of programs like Sammanit Jeevan in Nepal or elsewhere? Has any evaluation to date looked at the impact of an intervention that includes all three components? Please summarize existing evidence around these intervention activities as it relates to the target outcomes. Further and importantly, the study shows very mixed evidence on several of the outcomes, without any way to determine which components of the study might have contributed to the negative consequences (increase in suicide ideation for older women, overall increase in sexual violence for younger women, etc.).

**Methods**

4. How was your sample size calculated?
5. Did all invited individuals agree to participate in the study? The flowchart in Figure 1 should be expanded to include the number of invited participants as a first row.
6. “Sessions run once weekly with separate same age-sex groups which came together for discussions every third session.” Does this mean there were four groups (100 younger women, 100 older women, 78 younger men, and 79 older men), or were these groups further broken down into several sessions? Please provide more details about the implementation of the program sessions and group makeup.
7. How long was each of the three intervention phases? Between what dates was the intervention implemented? Was the intervention still ongoing during the 6-month follow-up point of data collection? Please clarify the timeline in the text and in Figure 1.
8. Were female interviewers used for female participants?
9. “Data was collected on the implementation of IGAs per family.” Was this data collected as part of the in-depth interviews? The surveys? Additional data collection? Please elaborate.
10. Given attrition, particularly among men, how was non-response addressed in the analysis? Did the authors use imputation? Was any analysis conducted to assess whether missing data was associated with outcomes of interest at earlier time points. I highly recommend you assess whether attrition is associated with these outcomes or, at the very least, discuss the implications of this in the limitations/discussion section.

**Results**

11. There is a Table 1 and Figure 1 in the methods section as well as the Results section. Please check the Table and Figure labels and references in text.
12. Table 1 in the Results section. I would suggest separating the summary statistics for the four main groups in the sample: younger women, older women, younger men, and older men.
13. Additionally, is it possible to report past-year IPV rather than lifetime, given that past-year is the timeframe for the primary outcomes?
14. Table 1. Am I reading correctly that 14% of women report experiencing sexual IPV and 0.7% of men report perpetrating?
15. The intervention is framed in the Background section as intended to target outcomes for young married women; however, the results for women are presented altogether (for young and older women combined). Is there a reason the authors decided to present the results in this way? All relevant outcomes and trends should be presented for the young married women’s group separately as well. For example, in Table 2, it is not clear if the increase in income for women is found in both the younger and older groups (and this distinction seemingly matters given the justification for the intervention). The same comment for the first panel in Table 3 as well.

**Discussion**

16. The increase in suicide ideation and self-reported impairment for the older female cohort is a worrisome finding. What do you attribute this increase to and how might this be mitigated in future iterations of the intervention?
17. There is only brief mention of lack of control group as a limitation. The authors should also discuss the self-reported nature of the outcomes, small sample size, and potentially biased attrition (as mentioned in comment #10) as limitations of this study.
18. My primary concern with the discussion section is the conclusion drawn in the first sentence: “Our findings provide considerable support for the Sammanit Jeevan whole family approach to reducing intimate partner violence, changing inequitable gender norms and economically empowering young women.” While the study did find improvement for some outcomes, *several* adverse effects were also observed, such as a decline in self-reported health, increase in suicide ideation for older women, increase in frequency of quarreling (as reported by women), increase in IPV for older women, increase in sexual violence perpetration by men, among several others. While increased comfort with reporting incidents of IPV may explain the increase in violence exposure, further qualitative research is needed to explore the extent to which this explanation accounts for the observed increase. Further, this likely does not explain the increase in suicide ideation and other negative outcomes for older women. The discussion section needs to more accurately discuss the potential adverse impacts of this intervention, how and which intervention activities might have contributed to these outcomes, and future implementation of the intervention might avoid these outcomes. Further, given all of this, I am not comfortable with the conclusion outlined in the first sentence of the Discussion.

**Abstract**

1. Please add the study setting to the abstract
2. The stats provided in parentheses are not clear- is this a range? A change from X to Y?
