# Supplementary material for: “I got courage from knowing that even a daughter-in-law can earn her living”: Mixed methods evaluation of a family-centred intervention to prevent violence against women and girls in Nepal
Source: PLoS One. 2020 May 19;15(5):e0232256. doi: 10.1371/journal.pone.0232256 (PMC7237029; doi:10.1371/journal.pone.0232256)
Supplement: Attachment — Submitted filename: response to reviewers_25458.docx [file pone.0232256.s002.docx]

**Reviewer#1**

**Abstract**

1. Background: I would remove the funding source (DFID What Works) to

save word count to bolster other sections. "young married women" is

repeated twice so removal of one reference would be preferred.

This has been addressed in page 2.

2. Results: The results are unclear as the measures are not specified. For

example, frequency changes in 'women's earnings' and 'savings' is not

clear until the reader reviews the measures table. Clarification of the

gender attitudes and perceptions of husband's control is needed for

whether the authors are referring to young women's changes or all

women's, including mothers-in-law.

The tables have been revised and now

include separate results for young women

and older women and these are reflected

in the abstract as well.

**Background**

Overall, the background is well written and it is appreciated that the

authors include customary practices as well as legal statutes in the

description. However, in the first paragraph, the authors write 'Nepal's

IPV prevalence is at least as high as other South Asian countries' but it is

unclear what the general trend is in other South Asian countries, so it is

recommended to include this information.

The trends of IPV in South Asia and

neighbouring countries has been written

into the first two paragraphs of this

section in page 3.

**Methods**

1. VDC is used in the participant section before it is defined. This has been corrected. The full definition

is provided under the Participant section

in page 5.

2. Further description of how the authors accounted for ethical

considerations when interviewing both men and women in the same

home about IPV perpetration and victimization, respectively. Typically,

this is not common in IPV research due to ethical concerns and potential

backlash of men. Were any additional steps taken?

This comment has been addressed, and

we added that the study was described as

a health and family life and relationships

project with an intervention to strengthen

relationships and livelihoods. We also

interviewed at different times and in

privacy, and family members were

encouraged not to discuss the interviews,

and none of the young married women

reported adverse reactions from their

husbands or in-laws after the interviews.

We also encouraged families not to

Cover Letter revision

**THE SOUTH AFRICAN MEDICAL RESEARCH COUNCIL**

1 Soutpansberg Road, Pretoria 0002 | Private Bag X385, Pretoria 0001, South Africa

Tel: +27 (0)12 339 8526 | Fax: +27 (0)12 339 8582 | Web: www.samrc.ac.za

GENDER & HEALTH RESEARCH

UNIT

discuss their interviews as everyone

would have their turn. See page 10.

3. The HFIAS is typically nine items. Description is needed as to which

items were included and why a smaller number was selected.

The HFIAS scale was adapted for different

settings in the What Works global

programme and the scale was also used to

make comparability in analysis possible

across the different settings.

4. Were the husband's family living with the index younger married

women? It is unclear if this was an inclusion criteria.

This has been addressed and inclusion

criteria is explained in page 5.

5. A sentence should be added on literacy levels of participants to

complete for signing informed consent, especially as more than half of

women reported no education. Was this a written or oral consent?

Participants who were could not write

were asked to make their mark on the

consent forms, after the study was

thoroughly explained and their questions

responded to satisfactorily. We have

recorded this as signed informed consent

to indicate that consent forms were

signed if participants consented to

participate. See page 10.

**Results**

1. In the second paragraph of the results, it is written 'women were more

likely to have been exposed to IPV in their lifetime compared to men'.

Given the following sentence includes statistics, this sentence is

recommended for removal or revision as it is not clear whether one is

referring to victimization or perpetration.

We deleted the sentence in page 11.

2. An interesting finding is that older women's report of their own

mother-in-law relationship gets worse is not addressed in the discussion.

Insights into this change should be included.

The worsening relations with older

women’s mothers-in-law is now discussed

in the discussion section in pages 19-20.

**Discussion**

1. The first paragraph states support for the intervention reducing IPV,

but this seems a stretch interpretation as only physical IPV among

younger women was reduced, with null or increased findings for other

forms of IPV, particularly for older women. Rephrasing this conclusion

would be recommended.

We have edited the discussion section to

reflect the reviewer’s comments but also

explain the effects of measurement error

on the IPV measures. See page 17-20.

2. The paragraph describing the increase in IPV being related to underreporting

at baseline is possible, but it does not address why IPV had

gone down at the midpoint periods of data collection for a few forms of

IPV. An additional alternate interpretation is whether the intervention

reduced men's migratory practices for work. If increased economic

wellbeing could lead to a reduction in this, it is possible men were home

more to perpetrate IPV at endline. If this, or other alternate explanations

seem viable, it should be included in this paragraph.

The IPV measures at midline were not

affected by migratory practices much as

they were impacted measurement error.

We edited the discussion to explain

measurement error, which was not

adequately explained in the original

manuscript. . See page 17-20.

3. The sentence on alcohol use seems out of place and additional

information could be added as to what parts of the intervention

addressed alcohol use.

More interpretation of the role of men’s

alcohol drinking has been included on

pages 18 and 19.

**REVIEWERS#2**

Reviewer #2: Thank you for providing me with the opportunity to review

this article. The authors discuss the evaluation of three-pronged

intervention to reduce violence against women and improve their

economic well-being. The article is well-written and easy to follow, and

Thank you for the comments. We have

revised the paper to reflect the negative

findings as well, see the Discussion section

in its entirety from page 17 to 20.

**THE SOUTH AFRICAN MEDICAL RESEARCH COUNCIL**

1 Soutpansberg Road, Pretoria 0002 | Private Bag X385, Pretoria 0001, South Africa

Tel: +27 (0)12 339 8526 | Fax: +27 (0)12 339 8582 | Web: www.samrc.ac.za

GENDER & HEALTH RESEARCH

UNIT

the findings are important to share with the development community.

However, in addition to the areas for improvement outlined below, I am

concerned with the lack of attention paid to the negative outcomes

observed in the findings. The authors conclude, “Our findings provide

considerable support for the Sammanit Jeevan whole family approach to

reducing intimate partner violence, changing inequitable gender norms

and economically empowering young women”, yet several adverse

effects were also observed, such as a decline in self-reported health,

increase in suicide ideation for older women, increase in frequency of

quarreling (as reported by women), increase in IPV for older women,

increase in sexual violence perpetration by men, among several others. In

addition to addressing the comments below, I would highly recommend

that the authors devote considerable attention to these negative findings

in the discussion section (in addition to amending the conclusion given

above).

**Abstract**

1. Please add the study setting to the abstract Setting has been included in the abstract

in page 2

2. The stats provided in parentheses are not clear- is this a range? A

change from X to Y?

The statistics have clarified in page 2.

Grammatical edits to the text are also needed. Grammatical errors have been reduced on

the manuscript as best we could.

**Background**

1. The third paragraph does an excellent job of explicating the factors

contributing to young married women’s experiences of violence as

perpetrated by their mothers-in-law and how these factors are linked to

gender inequality and patriarchal family structures. However, the

majority of the Background is focused on this topic, which is not the

primary outcome of the intervention as stated in Table 1. I believe the

second paragraph could be strengthened by expanding on the

mechanisms that foster IPV in this setting, rather than simply stating

various characteristics found to be associated with IPV in Nepal.

We have addressed these comments

throughout the Background section to

explicate other factors mechanisms that

foster IPV in this setting. See pages 3-5.

2. Given that primary outcomes also include economic- and incomebased

indicators, the Background section should provide background on

women’s labor force participation and income decision-making in this

context, including barriers to labor participation and the underlying

gendered power dynamics that inherently marginalize women in this

area. Such discussion of this literature will make clear for the reader why

the intervention includes the three primary components of gender

transformative norms, economic empowerment, and IGAs support.

The literature on the economic and

income related indicators have been

included in the paper, to illustrate the

positioning of women’s labour in Nepal in

page 4.

3. What evidence currently exists around the effectiveness of programs

like Sammanit Jeevan in Nepal or elsewhere? Has any evaluation to date

looked at the impact of an intervention that includes all three

components? Please summarize existing evidence around these

intervention activities as it relates to the target outcomes. Further and

importantly, the study shows very mixed evidence on several of the

outcomes, without any way to determine which components of the study

might have contributed to the negative consequences (increase in suicide

The information on an evaluation of a

similar intervention has been mentioned

in the discussion session, see pages 17 to

20.

**THE SOUTH AFRICAN MEDICAL RESEARCH COUNCIL**

1 Soutpansberg Road, Pretoria 0002 | Private Bag X385, Pretoria 0001, South Africa

Tel: +27 (0)12 339 8526 | Fax: +27 (0)12 339 8582 | Web: www.samrc.ac.za

GENDER & HEALTH RESEARCH

UNIT

ideation for older women, overall increase in sexual violence for younger

women, etc.).

**Methods**

4. How was your sample size calculated? The description for the sample size

calculation has been added on page 5

under Participation subsection.

5. Did all invited individuals agree to participate in the study? The

flowchart in Figure 1 should be expanded to include the number of

invited participants as a first row.

Enrolment of participants has been

revised and explained in pages 5 to 6 and

the flowchart updated.

6. “Sessions run once weekly with separate same age-sex groups which

came together for discussions every third session.” Does this mean there

were four groups (100 younger women, 100 older women, 78 younger

men, and 79 older men), or were these groups further broken down into

several sessions? Please provide more details about the implementation

of the program sessions and group makeup.

The groups have been explained in page 7.

7. How long was each of the three intervention phases? Between what

dates was the intervention implemented? Was the intervention still

ongoing during the 6-month follow-up point of data collection? Please

clarify the timeline in the text and in Figure 1.

The intervention phases have been

explained in more detail. The flow chart

has also been updated to reflect the

period of data collection. See pages 6 and

7.

8. Were female interviewers used for female participants?

Yes, this has been explained in page 7.

9. “Data was collected on the implementation of IGAs per family.” Was

this data collected as part of the in-depth interviews? The surveys?

Additional data collection? Please elaborate.

This has been explained in page 9.

10. Given attrition, particularly among men, how was non-response

addressed in the analysis? Did the authors use imputation? Was any

analysis conducted to assess whether missing data was associated with

outcomes of interest at earlier time points. I highly recommend you

assess whether attrition is associated with these outcomes or, at the very

least, discuss the implications of this in the limitations/discussion section.

The attrition among men was expected

due migration for work among younger

men. However, our analysis showed no

association between any of the outcomes

at baseline (IPV perpetration, income,

savings, earnings, mental health

outcomes) and attrition.

Thus we assumed non-availability at 18m

was random and no imputation was done

for missing participants at 18 months.

We have added a statement in the

limitations data analysis section on this.

See pages 9 to 10.

**Results**

11. There is a Table 1 and Figure 1 in the methods section as well as the

Results section. Please check the Table and Figure labels and references

in text.

This has been corrected throughout the

manuscript.

12. Table 1 in the Results section. I would suggest separating the

summary statistics for the four main groups in the sample: younger

women, older women, younger men, and older men.

This has been done as suggested see

tables from 2 to 4, IPV table has remained

the same.

13. Additionally, is it possible to report past-year IPV rather than lifetime,

given that past-year is the timeframe for the primary outcomes?

We have added past year prevalence to

the Table.

14. Table 1. Am I reading correctly that 14% of women report

experiencing sexual IPV and 0.7% of men report perpetrating?

Yes but it is now table 2.

**THE SOUTH AFRICAN MEDICAL RESEARCH COUNCIL**

1 Soutpansberg Road, Pretoria 0002 | Private Bag X385, Pretoria 0001, South Africa

Tel: +27 (0)12 339 8526 | Fax: +27 (0)12 339 8582 | Web: www.samrc.ac.za

GENDER & HEALTH RESEARCH

UNIT

15. The intervention is framed in the Background section as intended to

target outcomes for young married women; however, the results for

women are presented altogether (for young and older women

combined). Is there a reason the authors decided to present the results in

this way? All relevant outcomes and trends should be presented for the

young married women’s group separately as well. For example, in Table

2, it is not clear if the increase in income for women is found in both the

younger and older groups (and this distinction seemingly matters given

the justification for the intervention). The same comment for the first

panel in Table 3 as well.

We have presented the outcomes for

young women and older women

separately as suggested.

**Discussion**

16. The increase in suicide ideation and self-reported impairment for the

older female cohort is a worrisome finding. What do you attribute this

increase to and how might this be mitigated in future iterations of the

intervention?

A paragraph to explain the findings on

older women has been included in page

19-20.

17. There is only brief mention of lack of control group as a limitation.

The authors should also discuss the self-reported nature of the outcomes,

small sample size, and potentially biased attrition (as mentioned in

comment #10) as limitations of this study.

A limitations section has been included in

the paper in page 20.

18. My primary concern with the discussion section is the conclusion

drawn in the first sentence: “Our findings provide considerable support

for the Sammanit Jeevan whole family approach to reducing intimate

partner violence, changing inequitable gender norms and economically

empowering young women.” While the study did find improvement for

some outcomes, several adverse effects were also observed, such as a

decline in self-reported health, increase in suicide ideation for older

women, increase in frequency of quarreling (as reported by women),

increase in IPV for older women, increase in sexual violence perpetration

by men, among several others. While increased comfort with reporting

incidents of IPV may explain the increase in violence exposure, further

qualitative research is needed to explore the extent to which this

explanation accounts for the observed increase. Further, this likely does

not explain the increase in suicide ideation and other negative outcomes

for older women. The discussion section needs to more accurately discuss

the potential adverse impacts of this intervention, how and which

intervention activities might have contributed to these outcomes, and

future implementation of the intervention might avoid these outcomes.

Further, given all of this, I am not comfortable with the conclusion

outlined in the first sentence of the Discussion.

We have revised the discussion section to

reflect these comments and have further

interpreted all the results.

**THE SOUTH AFRICAN MEDICAL RESEARCH COUNCIL**

1 Soutpansberg Road, Pretoria 0002 | Private Bag X385, Pretoria 0001, South Africa

Tel: +27 (0)12 339 8526 | Fax: +27 (0)12 339 8582 | Web: www.samrc.ac.za

GENDER & HEALTH RESEARCH

UNIT

We trust that these revisions will be satisfactory.

**Nwabisa Shai***, PhD*

Specialist Scientist: Gender and Health Research Unit,

South African Medical Research Council
