# Supplementary material for: “I got courage from knowing that even a daughter-in-law can earn her living”: Mixed methods evaluation of a family-centred intervention to prevent violence against women and girls in Nepal
Source: PLoS One. 2020 May 19;15(5):e0232256. doi: 10.1371/journal.pone.0232256 (PMC7237029; doi:10.1371/journal.pone.0232256)
Supplement: Attachment — Submitted filename: Response to Reviewers Comments 16032020 final .docx [file pone.0232256.s003.docx]

**Reviewers’ comments**

**Comments to the Author**

Reviewer #2: The authors have done a very satisfactory job of addressing my comments and I recommend this manuscript for acceptance. However, given that PLOS ONE does not copyedit accepted manuscripts, the manuscript might benefit from one more round of editing for grammatical errors.

Reviewer #3: This is a well written manuscript that details the effectiveness of the Samaanit Jeevan intervention in Nepal. There are a few limitations that dampen my enthusiasm for this manuscript, and one specifically that needs to be addressed (A) without some type of comparison group (formal control arm, analytic control methods, sensitivity analyses), it is impossible to evaluate if the program was effective or if this study documents an artifact of the historical context where individuals and community values are changing; (B) insufficient attention to sample size, especially with diverse ethnic groups in region; and (C) intervention design has some key limitations – in addition to comparison group issue - including discussions on how other family members participated in programming. I elaborate on all points in my comments for the authors below, however sufficient detail was provided by previous reviewers and thus I make an attempt to avoid double commenting on those items.

# Response to reviewers’ comments

| **Reviewers’ comments** | **Authors’ responses** |
| --- | --- |
| Reviewer 2:  The manuscript might benefit from one more round of editing for grammatical errors. | This has been addressed. Thank you. |
| Reviewer 3 comments: |  |
| Reviewer #3: This is a well written manuscript that details the effectiveness of the Samaanit Jeevan intervention in Nepal. There are a few limitations that dampen my enthusiasm for this manuscript, and one specifically that needs to be addressed (A) without some type of comparison group (formal control arm, analytic control methods, sensitivity analyses), it is impossible to evaluate if the program was effective or if this study documents an artifact of the historical context where individuals and community values are changing; (B) insufficient attention to sample size, especially with diverse ethnic groups in region; and (C) intervention design has some key limitations – in addition to comparison group issue - including discussions on how other family members participated in programming. I elaborate on all points in my comments for the authors below, however sufficient detail was provided by previous reviewers and thus I make an attempt to avoid double commenting on those items. | |
| (A) Lack of comparison group. While highlighted by the authors as a limitation, this design flaw was not addressed analytically at all. Effectiveness cannot be established without comparing findings to those who did not receive the intervention, especially in communities and contexts that are changing as rapidly as modern Nepali villages are changing. Additional work with inverse probability weighting or sensitivity analyses could have at least worked towards addressing these issues. However, as written, the basic conclusions around effectiveness lack evidence to support them. | Our study is a proof of concept that sought to test a new intervention in a new research setting. We used a single-arm design to intervention evaluation to determine preliminary effects of the intervention, assess initial reception of the intervention by participants, and implementation requirements before running it through a randomised controlled trial, if the pilot was successful. A number of studies in the field of GBV prevention have used single arm evaluations to pilot test their newly designed intervention. For instance, Jewkes, Gibbs et al (2014) obtained preliminary data that helped to determine the effectiveness of combining a well-established gender transformative programme (Stepping Stones) with a new livelihoods strengthening component (Creating Futures) through an evaluation with no control arm (JEWKES, R., GIBBS, A., JAMA-SHAI, N., WILLAN, S., MISSELHORN, A., MUSHINGA, M., WASHINGTON, L., MBATHA, N. & SKIWEYIYA, Y. 2014. Stepping Stones and Creating Futures intervention: shortened interrupted time series evaluation of a behavioural and structural health promotion and violence prevention intervention for young people in informal settlements in Durban, South Africa. *BMC Public Health,* 14**,** 1325.). This study was useful in establishing the suitability of combining the two different components and this was later evaluated through an RCT, the results of which built on the preliminary evaluation findings (GIBBS, A., WASHINGTON, L., ABDELATIF, N., CHIRWA, E., WILLAN, S., SHAI, N., SIKWEYIYA, Y., MKHWANAZI, S., NTINI, N. & JEWKES, R. 2020. Stepping Stones and Creating Futures Intervention to Prevent Intimate Partner Violence Among Young People: Cluster Randomized Controlled Trial. *Journal of Adolescent Health,* 66**,** 323-335.). Another evaluation study by Corboz and colleagues in Afghanistan used a single arm approach and established some promising evidence that peace building and community social norm change interventions in conflict settings can be effective in reducing peer violence victimization and peer violence perpetration among secondary school pupils, and corporal punishment by teachers (CORBOZ, J., SIDDIQ, W., HEMAT, O., CHIRWA, E. D. & JEWKES, R. 2019. What works to prevent violence against children in Afghanistan? Findings of an interrupted time series evaluation of a school-based peace education and community social norms change intervention in Afghanistan. *PLOS ONE,* 14**,** e0220614.). This study was useful in providing preliminary evidence of viability of peacebuilding models used by a local NGO in Afghanistan. This study was published in Plos One.  We understand that RCTs are gold standard for effectiveness testing, but researchers are advised not to run them without initial indications of potential effectiveness of the intervention. RCTs are expensive, time consuming and inflexible and they are not appropriate for the stage when information from the evaluation is needed in a short period of time to enable adjustments to optimise an intervention. It is much more cost-effective to ensure there is some merit for testing interventions before major evaluations are conducted. These studies which show preliminary evidence of promise are generally referred to as Phase 2 trials in clinical research, although this terminology is less often used in social intervention evaluations. It is important to publish findings from single arm Phase II studies as researchers about the information emerging through the process of the evaluation, mindful that the lessons about novel programmes do not only come from RCTs. In clinical research, paper have been published about how to conduct Phase 2 trials for example:   A. MANDREKAR, S. J. & SARGENT, D. J. 2010. Randomized phase II trials: time for a new era in clinical trial design. *Journal of thoracic oncology: official publication of the International Association for the Study of Lung Cancer,* 5, 932-934. B. SHIM, S. R., KIM, J. H., CHOI, H., LEE, W. J., KIM, H. J., BAE, M. Y., HWANG, S. D., KIM, K. H., BAE, J. H. & YOON, S. J. 2015. General effect of low-dose tamsulosin (0.2 mg) as a first-line treatment for lower urinary tract symptoms associated with benign prostatic hyperplasia: a systematic review and meta-analysis. *Curr Med Res Opin,* 31, 353-65. C. VAN NORMAN, G. A. 2019. Phase II Trials in Drug Development and Adaptive Trial Design. *JACC. Basic to translational science,* 4, 428-437. D. ZHENG, L., ROSENKRANZ, S. L., TAIWO, B., PARA, M. F., ERON, J. J., JR. & HUGHES, M. D. 2013. The design of single-arm clinical trials of combination antiretroviral regimens for treatment-naive HIV-infected patients. *AIDS research and human retroviruses,* 29**,** 652-657.     Since GBV is very serious problem affecting women and girls many prevention interventions exist and often they have not been evaluated. By publishing this paper, we hope to contribute towards developing an evidence base and providing information about how we have approached this proof of concept research may be of value to others wishing to do likewise.  Also see our additional edits on the limitations section in page 17. |
| (B) Further discussion of sample size considerations would also strengthen this manuscript. Given the different ethnicities presented in the study population and the heterogeneous nature of attitudes towards IPV, further discussion and perhaps sensitivity analyses by primary ethnic groups is warranted. | We worked in a small area of Nepal, and our intervention covered only 6 villages in the 2 VDCs and these were all quite close by. This did not enable us to reach very diverse ethnic groups. There was some ethnic diversity in the communities, and particular caste diversity, but through our research we found that statistically there was not much difference in how members from different castes and ethnic groups responded to the intervention, this is reported in the study (). In addition, since this is a proof of concept study, the design was not intended to be powered to conduct the type of subgroup analysis the reviewer has raised. These sub-group analyses will be explored in more depth in a bigger RCT with bigger sample size, should this be conducted.  Researchers in the oncology field have interrogated the use of phase II trials with no control arms and argued that these trials are designed to identify promising interventions that can be tested further in phase III trials. Phase II trials do not need to have a control. They acknowledge that these studies are seldom fully powered as the focus is on gaining insights about the intervention and sufficient evidence to justify a RCT trial (MANDREKAR, S. J. & SARGENT, D. J. 2010. Randomized phase II trials: time for a new era in clinical trial design. *Journal of thoracic oncology: official publication of the International Association for the Study of Lung Cancer,* 5, 932-934.). New interventions also require initial testing for general effects and other researchers in this field have used this single arm phase II trial design to demonstrate this and determine safety of cancer treatment (SHIM, S. R., KIM, J. H., CHOI, H., LEE, W. J., KIM, H. J., BAE, M. Y., HWANG, S. D., KIM, K. H., BAE, J. H. & YOON, S. J. 2015. General effect of low-dose tamsulosin (0.2 mg) as a first-line treatment for lower urinary tract symptoms associated with benign prostatic hyperplasia: a systematic review and meta-analysis. *Curr Med Res Opin,* 31, 353-65). Phase II trials with no control arm are seen as a cost-effective way of determining the usefulness of certain cancer treatment and predicting success in Phase III drug trials and can shorten overall drug development time which is valuable to the field (VAN NORMAN, G. A. 2019. Phase II Trials in Drug Development and Adaptive Trial Design. *JACC. Basic to translational science,* 4, 428-437.).  In the antiretroviral testing field, Zheng and colleagues further expound that phase II trials with no control arms can be useful to establish initial estimates of efficacy and safety (ZHENG, L., ROSENKRANZ, S. L., TAIWO, B., PARA, M. F., ERON, J. J., JR. & HUGHES, M. D. 2013. The design of single-arm clinical trials of combination antiretroviral regimens for treatment-naive HIV-infected patients. *AIDS research and human retroviruses,* 29**,** 652-657.).  Safety is key for GBV programming, for instance, our study also sought to determine the safety of our intervention in context where young married women’s movements were restricted and found that the success of the young women led interventions was in part to change in attitudes about women’s movements. |
| (C) The overall intervention design has a few components that are further troubling, even if the above two issues are/were addressed. For example, there is a long history in Nepal of lower-powered individuals (be it by caste, ethnicity, or gender) being manipulated by socially proximal higher powered individuals. Further discussion into how ‘other family members’ who participated with the women were selected, their backgrounds, and the nature of their input, is essential to understand the true scope and impact of this intervention. | We were specific in our participant recruitment that we sought consent from the young married woman first, and then accessed her husband and his parents. Thus, we did not allow random individuals from a family to participate in the study or have a say in the young married women’s involvement in the study. We appreciate the reviewer’s concern, but we employed a strict recruitment strategy as we have explained it in the paper. |
